# Case Report: A patient with a novel heterozygous IRF8 variant with repeated infection and immune-mediated organ disease, but without disseminated mycobacterial disease despite BCG immunization

**DOI:** 10.3389/fimmu.2025.1654617

**Published:** 2025-10-28

**Authors:** Samuel C. C. Chiang, Erika Owsley, Ammar Husami, Nagako Akeno, Cristina M. Cobb, Li Yang, Rebecca A. Marsh, Kenneth A. Myers, Tamar S. Rubin

**Affiliations:** ^1^ Division of Bone Marrow Transplantation and Immune Deficiency, Cincinnati Children’s Hospital Medical Center, Cincinnati, OH, United States; ^2^ Department of Pediatrics, University of Cincinnati, Cincinnati, OH, United States; ^3^ Division of Human Genetics, Cincinnati Children’s Hospital Medical Center, Cincinnati, OH, United States; ^4^ Child Health and Human Development Program, Research Institute of the McGill University Health Center, Montreal, QC, Canada; ^5^ Division of Neurology, Department of Pediatrics, McGill University, Montreal Children’s Hospital, Montreal, QC, Canada; ^6^ Department of Neurology & Neurosurgery, McGill University, Montreal, QC, Canada; ^7^ Division of Pediatric Clinical Immunology and Allergy, Children’s Hospital Winnipeg, Winnipeg, MB, Canada; ^8^ Department of Pediatrics and Child Health, University of Manitoba, Winnipeg, MB, Canada

**Keywords:** case report, IRF8, liver failure, plasmacytoid DCs, inborn errors of immunity (IEI)

## Abstract

We describe a patient with a novel, *de novo* heterozygous *IRF8* variant (c.1182dup) who presented with viral and bacterial susceptibility, lymphoproliferation, and liver and lung diseases characteristically seen in patients with underlying inborn errors of immunity, but without disseminated mycobacterial disease, despite vaccination with Bacillus Calmette-Guérin (BCG), the live attenuated vaccine form of *Mycobacterium bovis*. Laboratory evaluation revealed an absence of circulating plasmacytoid dendritic cells (pDC) with poor IL-12p70 and IFN-γ secretion upon LPS stimulation, and poor IFN-γ secretion upon PHA stimulation. In contrast, another patient with a different novel, *de novo* heterozygous *IRF8* variant (c.10C>T) had milder early life infection susceptibility, but no lymphoproliferation, nor immune-mediated organ disease, and no prior exposure to BCG vaccine. They had a normal number of circulating pDC, and IL-12p70 production upon LPS stimulation that was no different compared to the mother who did not possess the *IRF8* variant, and with normal IFN-γ secretion upon PHA stimulation. Our findings support impaired *IRF8* function in the first patient, but less for the second patient. We propose that altered DC subsets and deficient cytokine production can assist with IRF8 VUS (variant of unknown significance) interrogation. This report expands current knowledge of mono-allelic human *IRF8* variants.

## Introduction

Interferon regulatory factors (IRF) regulate gene expression and immune responses to interferon, and possess N-terminus DNA binding domains (DBD), and C-terminus IRF-association domains (IAD), the latter allowing for dimerization and cofactor interaction (1–3). Mouse models have elucidated the functional roles of interferon regulatory factor 8 (IRF8) in monocyte and dendritic cell differentiation, and inhibition of neutrophil development (1, 4, 5). Irf8 knockout mice show increased viral infections, reduced IL-12p40 and IFN-gamma production, neutrophil expansion and a blast crisis phenotype, and lower numbers of pDCs and CD8α+ mDCs, but not CD11b+DCs (6–8). Mice with IRF8 IAD mutations affecting dimerization retain monocytes, macrophages and pDCs, but develop a chronic myeloid leukemia-like disease and cannot produce IL-12 or IFN-gamma (9).

In mice, IRF8 promotes monocyte and dendritic cell differentiation via its interaction with PU.1 and induction of KLF4. Neutrophil development is hindered by interfering with the transcription factor CEBPA binding chromatin in monocyte-DC progenitors. IRF8 is also critical for pDC function, and terminal differentiation of cDC1 via BATF3 interaction (10, 11). It is essential for IL-12p35, Il-12p40 and IL-18 expression, and its absence impairs Th1 responses, increasing intracellular pathogen susceptibility in Irf8-/- mice (11–15).

Currently, nine human cases of IRF8-related disease are reported, five with autosomal recessive inheritance, and four with 3 *de novo* dominant negative variants, including a mother-child dyad. Two phenotypes emerge in these reports: an autosomal recessive severe immunodeficiency with impaired myeloid lineage, and a dominant negative form with decreased cDC2s and mycobacterial susceptibility (16). NK cell developmental and functional defects were additionally described in one autosomal recessive case (17).

The first *IRF8* deficiency patient reported had biallelic variants leading to disseminated BCG (Mendelian Susceptibility to Mycobacterial Disease (MSMD), and fungal infections (18, 19) They had deficient monocytes and dendritic cells with failure to produce either IL-12 or IFN- γ upon LPS stimulation (13, 20). Subsequent autosomal recessive IRF8 deficiency cases were also reported to have recurring viral infections, BCG susceptibility, absent or decreased monocyte and DC populations, and decreased IL-12 and IFN-γ production (21, 22). In contrast, dominant negative patients showed reduced CD1c+ DCs and IL-12 production, while also demonstrating MSMD (22). Recently, a dominant negative variant was described in mother and child, with decreased pDC and cDC1, but normal cytokine production.

We describe an individual with previously unreported heterozygous *IRF8* variant causing a frameshift mutation in the last exon, and cellular deficiencies consistent with previous reports, leading us to conclude the identified *IRF8* variant is likely pathogenic. We juxtapose this against a second patient with a different, also previously unreported heterozygous *IRF8* variant, with a less convincing clinical and immunologic presentation for inborn error of immunity (IEI), and where the cellular phenotype tested was unaltered. We propose that a combination of specific cellular testing could help interrogate *IRF8* variants of uncertain significance (VUS).

## Case description

### Patient 1

The patient was a female of mixed European and Indigenous background who received BCG vaccination at birth. She developed recurrent oto-sinopulmonary infections and chronic bilateral parotitis in her first year of life. By age 5 she developed bronchiectasis, and had repeatedly borderline sweat chloride results. She had Epstein-Barr virus infection by age 6, with high viral titers. She required recurring hospitalizations for pneumonia and bronchiectasis exacerbations, including mechanical ventilation at age 9 for complicated pneumonia. At this point she had hepatosplenomegaly and failure to thrive.

By age 14, she demonstrated a chronic cholestasis liver enzyme pattern, and exocrine pancreatic insufficiency, based on elevated stool fecal-elastase and diarrhea. Furthermore, she had generalized lymphadenopathy and malnutrition, with low weight and short stature. CFTR sequencing plus a ciliary dyskinesia gene panel were both negative, and nasal brushing electron microscopy did not support ciliary dyskinesia diagnosis either. Evaluation for Sjogren’s syndrome and sarcoidosis was negative. Axillary lymph node biopsy demonstrated non-caseating granulomatous inflammation, and liver biopsy showed chronic biliary obstruction with portal fibrosis. Repeated tissue and sputum cultures over years were negative for mycobacteria.

Immunologic evaluations demonstrated chronic mild neutrophilia and monocytosis, and chronic NK lymphopenia, with evolving T cell lymphopenia (**Table 1**) (23). She had a normal absolute number of B cells, and hypergammaglobulinemia. She had markedly reduced proportions of CD4+CD45RA+ and CD4+CD45RA+CD31+ T cells, and increased proportions of activated CD8+CD45RA+, with normal T cell proliferative responses to phytohemagglutinin (PHA), Concanavalin A, and pokeweed. She demonstrated specific responses to routine protein-based childhood immunizations such as mumps, and protective IgG titers following tetanus boosters. She was also able to demonstrate appropriate short-term response to 23-valent pneumococcal polysaccharide immunization. However, she did not demonstrate a protective response to diphtheria, measles or rubella, following childhood vaccination and diagnostic boosters, and her protective responses to childhood tetanus and pneumococcal conjugate vaccines appeared to wane quickly, as evidenced by non-protective levels on sampling after a short interval following immunization. The patient was not receiving any immunosuppression or other treatments that would have influenced the vaccine response. They were not receiving immune globulin replacement or other passive immunization at any time during their evaluations. Interestingly, she had persistent EBV viremia many years after initial infection. Trio exome sequencing was pursued, identifying several VUS in immune- related genes (**Table 2**).

**Table 1:** Select immune and infectious results at various ages for Patient 1 bearing c.1182dup (p.Glu395Arg fs*75), and for Patient 2 bearing c.10C>T.

Evaluated Parameter	P1 Age 9*	P1 Age 14	P1 Age 19	P1 Age 19	P2 age 4	P2 age 10	P2 age 12
Absolute neutrophil count	**58.79**	**15.51** (1.5 – 8.5)	**9.93** (1.8 -5.4)	**5.92** (1.8 – 5.4)			2.12
Absolute monocyte count		**2.13** (0 – 0.8)	**0.84** (0.3 – 0.8)	**0.84** (0.3 – 0.8)			0.55
CD3 (cells/mm3)	**2675** (1400-2000)	**766** (1000-2200)	**200** (668-2291)	**311** (668-2291)	2320	1132	
CD4 (cells/mm3)	**1588** (700-1100)	**452** (530 – 1300)	**126** (433-1692)	**235** (433-1692)	1311	678	
CD8 (cells/mm3)	**961** (600-900)	**244** (330-920)	**52** (147-1068)	**61** (147 – 1068)	760	348	
CD19 (cells/mm3)	**1379** (300-500)	**70** (110-570)	155 (79-574)	158 (79-574)	1050	470	
CD3-CD16+CD56+ (cells/mm3)	**125** (200-300)	**35** (70-480)	**19** (38-561)	41 (38 – 561)	527	146	
IgG (g/L)	**34.3**	**23.5**	14.7	15.7 (6.9- 16.2)	8.26	11.65	
IgA (g/L)	1.05	**0.48** (0.54 – 3.78)	**0.33** (0.7 – 3.5)	**0.38** (0.7 – 3.8)	**2.73** (0.38 – 2.39)	1.9	
IgM (g/L)	0.66	0.88	1.1	0.7 (0.6 – 2.6)	1.31	1.49	
IgE (IU/mL)	5	3	2	2 (0-100)	<5		
Rheumatoid factor (IU/mL)	**385**	**104** (0-2)					
EBV NAAT			**5.77 x 10^4 copies/mL**	**1.78 x 10^5 copies/mL**			undetected
EBV IgG	Positive		Positive				
EBV IgM	**Positive**		**Positive**				
Tetanus IgG (IU/mL)	**0.11**	0.53	0.39	2.718	0.2		
Diphtheria IgG (IU/mL)		**0.02**	**<0.01**	**0.035**	0.1		
Mumps IgM		**Positive**	**Positive**				
Mumps IgG			Positive				
Measles IgG			**Negative**				
Rubella IgG		Negative	**Negative**				
Pneumococcal capsular polysaccharide IgG (mg/L)		**2.672**		34.475			
Pneumococcal capsular polysaccharide IgG2 (mg/L)		**0.66**		6.814			
%CD4+CD45RA+			**9%** (12-70)				
%CD8+CD45RA+			**30%** (32– 94)				
%CD8+HLADR+CD28+			**49** (3- 38)				
%CD4+CD45RA+CD31+			**9.1**				
%IgM only memory B cells			**5.9** (0 – 5.3)	4.0 (0 – 5.3)			
%class switched memory B cells			**1.4** (2.3 – 26.5)	**1.1** (2.3 – 26.5)			
B cells with reduced CD21 expression			**15%**	2%			
%T follicular helper cells (CD4+CD45RA-CXCR5+)				**37** (10-29)			
Lymphocyte proliferation to antigens (PHA, PWM, ConA)			Normal				

*During hospital admission for pneumonia.

Results in bold indicate values beyond reference range.

**Table 2:** Variants reported for Patient 1.

Gene	Disorder	Mode of inheritance	Transcript	DNA variation	Reference
*BCL11B*	Immunodeficiency 49	Autosomal dominant	NM_022898.2	c.1308C>T (p.Gly436Gly) heterozygous	Rs745896590
*IRF8*	Immunodeficiency 32A, mycobacteriosis, autosomal dominant; Immunodeficiency 32B, monocyte and dendritic cell deficiency, autosomal recessive	Autosomal dominant; Autosomal recessive	NM_002163.2	c.1182dup (p.Glu395Argfs*75) heterozygous	Undocumented
*PKHD1*	Polycystic kidney disease 4, with or without hepatic disease	Autosomal recessive	NM_138694.3	c.6433C>A (p.Leu2145Ile) heterozygous	gnomAD Database
*SKIV2L*	Trichohepatoenteric syndrome 2	Autosomal recessive	NM_006929.4	c.2752G>A (p.Val918Ile) heterozygous	Rs148798682
*SMARCAL1*	Schimke immunoosseous dysplasia	Autosomal recessive	NM_014140.3	c.379G>C (p.Ala127Pro) heterozygous	Rs62178625

P1’s liver and lung diseases progressed, and she was referred for combined liver and lung transplant at age 19. A repeat lymph node biopsy was negative for malignancy and mycobacteria, with scant histiocyte aggregates, but no definite granulomas. Her liver disease progressed to fibrosis and cirrhosis, with portal hypertension, esophageal varices, and ascites. Liver imaging features were suggestive of nodular regenerative hyperplasia or lymphoproliferative disorder. Liver biopsy at age 14 noted focal periportal cholestasis, proliferation of cholangioles, and portal fibrosis, thought to represent chronic, likely incomplete, biliary obstruction, but without evidence of autoimmune hepatitis. Liver biopsy at age 19 noted chronic cholestasis with marked septal fibrosis (Stage 3-4/4), consistent with obstructive biliary tract disease. Portal tracts contained a mixed infiltrate of lymphocytes and plasma cells. There was a marked periportal cholestasis on Rhodamine strain, but no florid duct lesion of early primary biliary cirrhosis, and no sclerosing cholangitis-like periductal fibrosis. Her lung disease was consistent with granulomatous lymphocytic interstitial lung disease common based on constellation of lymphadenopathy, restrictive spirometry, immunodeficiency, cholestatic-fibrotic liver disease, and granuloma formation on prior lymph node biopsies, in addition to the known bronchiectatic changes.

**Figure 1:**
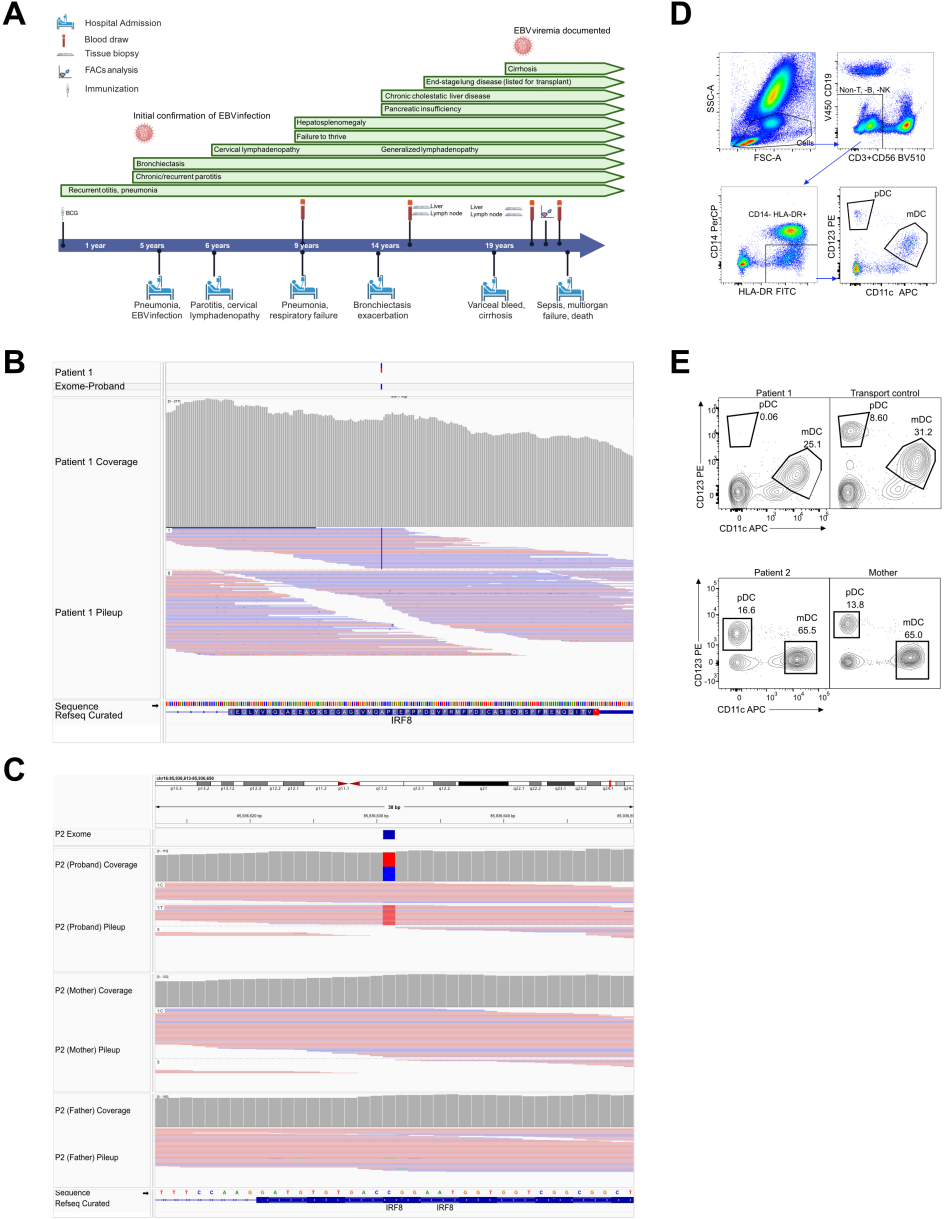
Patient timeline and molecular diagnosis. **(A)** Patient 1 timeline showing clinical presentation. **(B)** Visualization of the Patient 1 IRF8 frameshift duplication variant using IGV. Sequencing data aligned to the GRCh37/hg19 reference genome. The variant occurs on chromosome 16 at position 85,954,784 (chr16:85,954,784) within the IRF8 gene. The VCF track indicates a heterozygous duplication (G/GC) supported by high-quality read alignments and balanced allele depth (AD = 103 reference reads, 89 alternate reads). The read coverage track demonstrates consistent sequencing depth across the locus, while the alignment track shows both reference and duplication-supporting reads. According to HGVS nomenclature, this variant is described as NM_002163.4(IRF8):c.1177dup at the cDNA level, Chr16(GRCh37):g.85954784dup at the genomic level, and results in a frameshift at the protein level (p.Ala393Glyfs*77). This results in an elongated IRF8 protein with an altered C-terminus. **(C)** Visualization of the *de novo* c.10C>T (p.Arg4Trp) missense variant from Patient 2. **(D)** Gating sequence for the analysis of various DC subsets. **(E)** CD123 and CD11c DCs gated for both patients.

At age 19, more detailed immunophenotyping tests described below were performed. At her last assessment, she had multiple verrucous-appearing lesions (not biopsied) on hands, feet and eyelids. Unfortunately, she died of multiorgan failure and sepsis while awaiting lung-liver transplantation (**Figure 1A**).

### Patient 2

The patient is a 12-year-old female with gastroesophageal reflux, feeding difficulties, and failure to thrive from age 2 months. At 20 months old, she developed ketotic hypoglycemia, with recurrent episodes, eventually requiring gastrostomy insertion to support feeding as hypoglycemic events could occur with only brief periods of fasting. She was noted to have early childhood recurrent infections, but these spontaneously reduced in frequency by around age 9. Infections isolated included common respiratory viruses (adenovirus, rhinovirus, coronavirus OC43, RSV, parainfluenza, enterovirus, influenza A and B and human metapneumovirus), and at least once she was found to have mycoplasma pneumoniae infection and Enterobacter urinary tract infection. She experienced recurrent *C. difficile* associated diarrhea. She had attention deficit/hyperactivity disorder and mild global developmental impairment but was able to do schoolwork at appropriate grade level, with support. She also had mild weakness, fatigability, and hypotonia. Electromyography found myopathic changes in her left deltoid. Serum creatine kinase was normal, and a broad panel of metabolic testing was non-diagnostic, leading to biopsies of the duodenum, liver, and skeletal muscle at age 4. The latter showed low glycogen content, leading to the suspicion of a glycogen storage disorder, though genetic testing was negative for pathogenic variants in any known associated genes. There was no evidence of inflammation or vasculitis on skeletal muscle biopsy. Duodenal biopsy was notable for eosinophilic infiltrate in the lamina propria focally within the epithelium, with associated epithelioid histiocytic inflammation but without well-formed granulomata, ultimately felt to be in keeping with eosinophilic enteropathy. Stains for mycobacteria in the upper gastrointestinal biopsies were negative, as was targeted assessment for Langerhans cell histiocytosis. Clinical laboratory immune evaluations showed intermittent mild lymphopenia, and no evidence of chronic neutrophilia or monocytosis on serial testing. However, while she had a normal dendritic cell population, she did express immature monocytes. She made normal response to tetanus and diphtheria immunization at age 4 (**Table 1**).

## Methods

Consent from P1 was obtained under IRB 2008–0483 at Cincinnati Children’s Hospital and for P2 under IRB 2018–3937 at McGill University.

Detailed methodologies are provided in the supplemental document.

## Molecular diagnosis

In P1, the c.1182dup indel in exon 9/9 causes a frameshift at Glu395 replacing the native 27-aa C-terminus with a 75-aa neo-tail and introduces a new stop codon within the last coding exon. Under the canonical last-exon/50-nt rules, the transcript is predicted to escape nonsense mediated decay, suggesting production of an elongated IRF8 protein with an altered C-terminus (24–26). This variant was *de novo* for P1 and not found in GnomAD (**Figure 1B**). **Table 2** lists all variants reported.

For P2, a *de novo* heterozygous missense variant in *IRF8*, c.10C>T (p.Arg4Trp) was identified. This variant is present 3 times in gnomAD, and is predicted “disease causing” by MutationTaster and “probably damaging” (score 1.000) by Polyphen-2 (**Figure 1C**). Genetic testing also identified a heteroplasmic VUS in the mitochondrial genome gene *MT-TC*, (m.5819T>C), present in 5.3% of reads; the variant was also identified in P2’s mother, present in 1.5% of reads (27).

## Laboratory findings

P1 had chronic mild monocytosis (**Table 1**) and an absence of IL-3Ra/CD123 expressing DCs, while CD11c positive DCs were comparable to controls (**Figures 1D, E**). P2, run on a different occasion, showed DCs comparable to simultaneously-run controls (**Figure 1E**). P1 showed significantly elevated CD21low B cells (15%, **Table 1**). This is observed in immune deficiencies, especially those with autoimmunity as well as hypogammaglobulinemia, and have been suggested to indicate chronic activation of the adaptive immune system (28–31). This supports the postulation of an immune-mediated chronic disease in P1. P1 also showed reduced CD31+ recent thymic emigrant T cells (9%, **Table 1**) (32). These are highly proliferative and replenish the peripheral T cell pool. The significantly reduced level in P1 at that age indicates lowered thymic output, suggesting immune exhaustion (33, 34).

**Figure 2:**
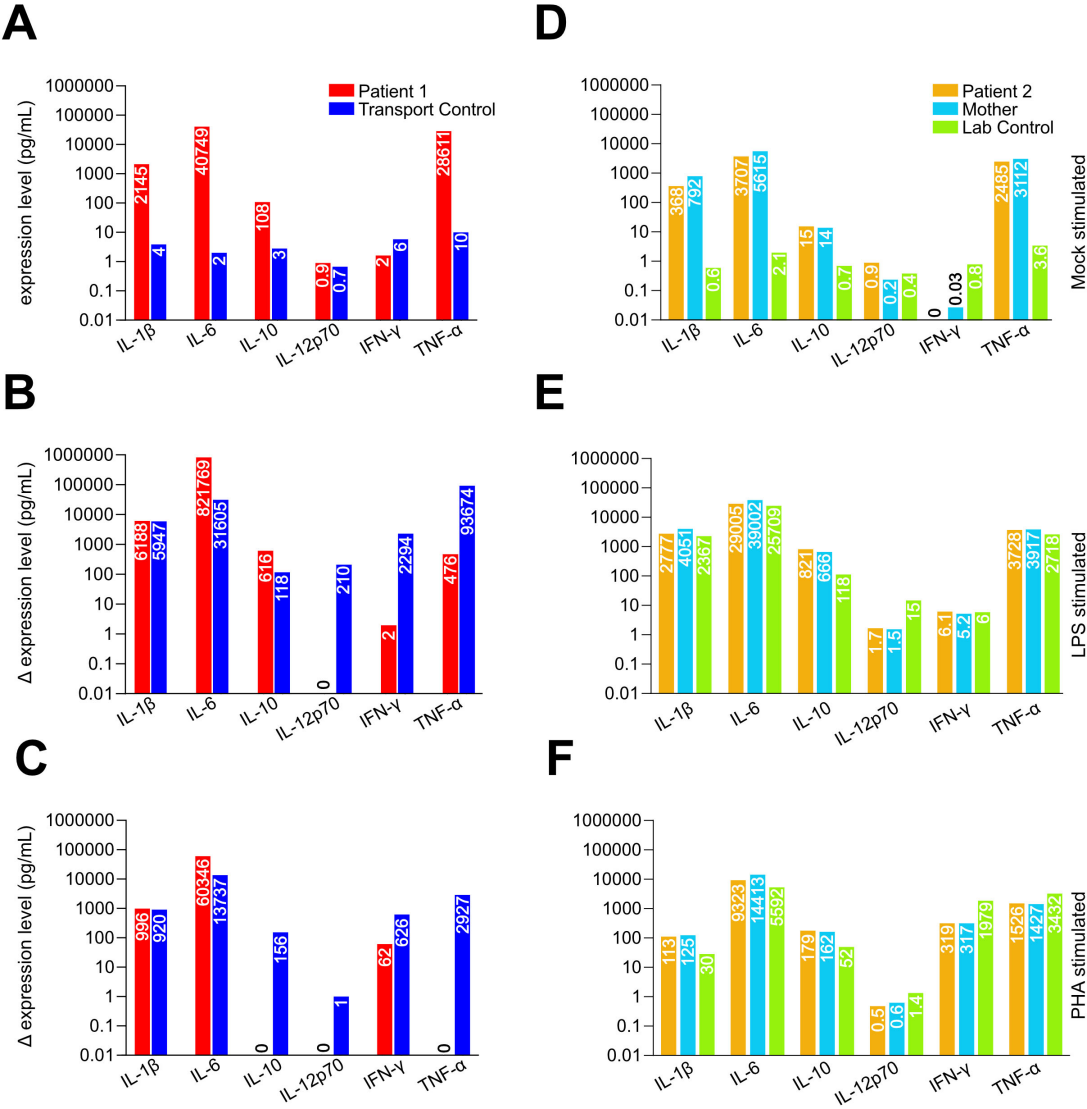
Bulk leukocyte cytokine response upon stimulation. Whole blood from **(A–C)** P1 and **(D–F)** P2 were stimulated with **(A, D)** PBS as control, **(B, E)** LPS, or **(C, F)** PHA. Patient 1 secretes IL-1β and IL-6 as expected upon stimulation but not IL-12p70. Levels of IFN-γ and TNF-α alpha are also lower compared to control. P2 and their mother displayed elevated resting state cytokines **(D)** but relatively comparable values after **(E)** LPS or **(F)** PHA stimulation, except for IL-12p70, which was significantly lower upon LPS stimulation. Values on the y-axis in log scale.

We tested functional cell responses via stimulation with LPS targeting monocytes and DCs, PHA targeting T cells, or mock stimulation. P1, P2, and the mother of P2 showed high cytokine levels when unstimulated, suggesting some pre-existing inflammatory condition, with increased baseline secretion of IL-1β, IL-6, IL-10, and TNF-α compared to control (**Figures 2A, D**). Upon LPS stimulation, P1 had comparable levels of IL-1β, IL-6, and IL-10 upregulation, reduced IFN- γ and TNF-α, and undetectable IL-12p70 (**Figure 2B**). PHA stimulation showed comparable IL-1β and IL-6 levels but low IFN-γ, and no increase in other readouts (**Figure 2C**). P2 and their mother showed reduced IL-12p70 levels when stimulated with LPS (**Figure 2E**). There were comparable values for all readouts upon PHA stimulation (**Figure 2F**).

## Discussion

We present two patients with novel monoallelic *IRF8* variants – c.1182dup (p.Glu395Argfs*75) and c.10C>T (p.Arg4Trp) – the former with a clinical presentation strongly suggestive of an underlying IEI, and the latter with a clinical presentation that was plausible for, but less strongly indicative of an IEI. We investigated whether these variants were the likely cause of the patients’ clinical features.

P1’s clinical and immunologic features strongly suggested a monogenic immune disorder, but clinical exome testing only revealed several VUS. Thus cellular studies were performed. We found increased neutrophils and monocytes, an absence of IL-3Ra/CD123 DCs, a high percentage of memory T cells, low recent thymic emigrants, expanded T follicular helper cells, and mostly naïve B cells with increased CD21low expression reminiscent of severe immune dysregulation patients (28, 35). All other lymphocyte populations studied were comparable to controls tested, including NK cells (17). Upon LPS stimulation, which targets monocytes and DCs, secreted IL-12p70, and, to a lesser degree, IFN- γ, were deficient (13, 14). Upon PHA stimulation, IL-10, IL-12p70, and TNF-α were not upregulated, indicating additional T cell response deficiencies, reminiscent of previously reported *IRF8* disease cases (20, 36).

The IRF8 transcription factor is essential for monocyte and DC development and function, and in this case, specifically CD123+ DCs (35, 37–39). AlphaFold/TED places the IRF8 DNA binding domain (aa 6–118) and IAD (aa 201–384) upstream of the variant. Prior work shows that this tail contributes to autoinhibitory control of the IAD and to partner-regulated chromatin engagement (18, 36). Consistent with this, C-terminal IRF8 mutations (e.g. G388S) and engineered C-tail extensions can diminish promoter binding, impair nuclear localization, and act dominant-negatively (24, 25, 40). We infer that p.Glu395Argfs*75 leads to loss of normal IRF8 function by disrupting C-terminal regulatory cues that govern cooperative binding with PU.1 and C/EBPα and the transcription of IRF8 targets. IRF members, together with PU.1, also upregulate B cell related genes such as CD20 and Ig light chain enhancers, possibly explaining the naïve B cell phenotype, impaired class switching, and reduced immunoglobulin secretion via CD70 surface expression (41–44). The total absence of pDCs in P1 was more reminiscent of the K108E, R83C/R291Q, or R111* autosomal recessive cases, than the milder T80A dominant negative cases, which demonstrated selective depletion of CD11c+CD1c+ DCs (18, 22, 45, 46).

Importantly, P1 received BCG vaccination in infancy and yet had no early or delayed adverse reaction to this live vaccine, with negative staining for acid-fast bacillus (AFB) on repeated tests, on multiple tissue samples. The monoallelic *IRF8* variant identified was therefore first viewed skeptically, as MSMD, including disseminated infection with minimally pathogenic mycobacteria like Bacillus Calmette-Guérin, was reported as a hallmark feature in cases of autosomal dominant and recessive IRF8 deficiency (18, 21). Indeed, environmental mycobacteria and BCG vaccines are usually eliminated effectively by macrophages activated by T-cells in healthy hosts, and previously, it was demonstrated that individuals with impaired mononuclear phagocyte development due to IRF8 variants were vulnerable to disseminated BCG in infancy or childhood, following vaccination at birth (18, 47). P2 had no history of having received BCG, nor did they present with evidence of mycobacterial disease. However, they did have a history of early life repeated viral infection, albeit less dramatic when compared to P1. P2 also presented with failure to thrive, with associated inflammatory changes in the duodenum involving epithelioid histiocytic inflammation without well-formed granulomas.

For P2, AlphaFold/TED places the IRF8 DNA-binding domain (aa 6–118) downstream of Arg4. The R4W substitution occurs at the N-terminus of the structured fold. The Arg to Trp change is predicted to perturb local electrostatics and DNA-contact geometry. Splicing prediction indicates altered exonic splicing regulatory elements with ~27% risk of impact (SPiP score 0.224) (48). Complementary in silico metrics show missense impact (CADD phred 23.7; MPA ‘moderate’), while splice-site–focused tools are largely negative (SpliceAI AG/AL/DG/DL all 0.00; AbSplice <0.01). The variant lies 11 bp from the acceptor of exon 2 and outside a UniProt-defined domain, extremely rare in gnomAD v4 (genomes ~1.3×10^−5^; exomes ~6.8×10^−7^; no homozygotes), and is currently a VUS in ClinVar. Taken together, c.10C>T could compromise IRF8 function through combined structural perturbation of the DBD N-terminus and carries a non-negligible risk of splicing dysregulation, but more data are needed to more definitively characterize the effects of this variant (49).

Significant heterogeneity in IRF8 patients has been documented, highlighted by a family with a significant reduction in NK cell number and function, and another with periodontal disease, indicating that there are yet to be determined roles of IRF8 (17, 40, 50, 51). While P1 did display reduced NK numbers over the years, NK phenotype was comparable to controls, although NK function was not tested. Dominant negative *IRF8* variants (c.1279dupT) have most recently also been noted to express reduced pDCs but normal cytokine expression (45). While we cannot rule out the missense variant effect in P2, with the available tests performed interrogating DC numbers and cytokine production after stimulation, we did not find changes to support immune impact of this variant similar to previously reported cases. However, P2’s early life history of repeated viral infections, failure to thrive, and poorly formed granulomatous changes on duodenal biopsy are notable, and do raise the question of an underlying IEI, perhaps related to their IRF8 variant causing an as of yet undefined immune impact.

In summary, we report two patients with novel heterozygous *de novo IRF8* variants (c.1182dup and c.10C>T). Data presented suggests the former variant is likely pathogenic, with features similar to both autosomal recessive and autosomal dominant IRF8 cases, including absent pDCs and lack of IL-12p70 production. However, the absence of mycobacterial infections despite mycobacterial exposure is unlike other reported cases thus far, and either indicates a wider disease heterogeneity or a confounding factor yet to be determined. P2, with less convincing symptoms of IEI and with apparently unaltered cellular phenotype, demonstrates the utility of performing combination tests to interrogate the pathogenicity of unknown variants. Further functional characterization of the two presented variants will be beneficial to better understand their impact on human immunity in our patients.

## Data Availability

The original contributions presented in the study are included in the article/**Supplementary Material**. Further inquiries can be directed to the corresponding author.
