# Supplementary material for: Case Report: A patient with a novel heterozygous IRF8 variant with repeated infection and immune-mediated organ disease, but without disseminated mycobacterial disease despite BCG immunization
Source: Front Immunol. 2025 Oct 28;16:1654617. doi: 10.3389/fimmu.2025.1654617 (PMC12602488; doi:10.3389/fimmu.2025.1654617)
Supplement: Supplementary file 1 [file DataSheet1.docx]

**Supplementary Methods**

**Case Report: A patient with a novel heterozygous IRF8 variant with repeated infection and immune-mediated organ disease, but without disseminated mycobacterial disease despite BCG immunization**

*Samuel C.C. Chiang, Erika Owsley, Ammar Husami, Nagako Akeno, Cristina Cobb, Li Yang, Rebecca A Marsh, Kenneth A Myers, Tamar Rubin*

FACS analysis for P1 was done September 3, 2021 when they were 19 years of age. While lab results presented in Table 1 were not run on the same day as the FACS analysis, they were done at the same age (June 2021 and September 15, 2021), and when the patient was not acutely ill with infection such as pneumonia. A timeline for P1 has been included as Figure 1A. Clinical immune evaluations were drawn for P2 at ages 4, 10 and 12 (November 2017, September 2022 and November 2024), at different times than the FACs analysis.

Dendritic Cell Phenotyping

Dendritic cells were examined for both patients as reported in Figure 1E.

Whole blood was stained with HLA-DR FITC (#340688, BD Bioscience), CD11c APC (#340714, BD Bioscience), CD3 BV510 (#317332, BioLegend), CD19 V450 (#644492, BD Bioscience), CD56 BV510 (#563041, BD Bioscience), CD123 PE (#340545, BD Bioscience), and CD14 PerCP (#340585, BD Bioscience). Red blood cells were lysed (BD FACS Lysing Solution, #349202) then washed once in PBS before being acquired on a BD FACSLyric cytometer and analyzed by FlowJo v10. Gating sequence outlined in Figure 1D.

Cytokine production

Cell stimulation was performed for the cytokine production test reported in Figure 2.

Whole blood was diluted 1:3 in RPMI supplemented with 2% human serum (H3667, Sigma) then stimulated 13 hours with either lipopolysaccharide (tlrl-3pelps, InvivoGen) at a final concentration of 1μg/mL, phytohemagglutinin (L9017, Sigma) at 10μg/mL, or mock stimulated with PBS. Cytokines were quantified using Ella (BioTechne) using multiplex cartridges for IL1β, IL-6, IL-10, IL-12p70, IFN-γ, and TNF-α.

Exome Sequencing

Sample from P1 was sent to Prevention Genetics (Marshfield, WI) for singleton sequencing. P2 was sent to GeneDX (Gaithersbyrg, MD). Pathogenic variants were identified based on the Human Gene Mutation Database (HGMD® Professional, QIAGEN®). [1] Variants were annotated and filtered based on ClinVar Pathogenic mutations. The read alignments were inspected using Integrated Genome Viewer. [2] The IGV screenshots are shown in Figure 1 B-C.

For P1, DNA extracted from whole blood in a clinical lab, and then frozen, was used for the singleton sequencing. Exome sequencing was performed on an Illumina NovaSeq platform using paired-end sequencing (2 × 100–150 bp reads). Raw sequencing reads were aligned to the human reference genome (hg19) using the BWA-MEM algorithm (v0.7.x). Aligned reads were processed with samtools (v1.11) for sorting and indexing. Read mapping quality was assessed through standard GATK metrics, including mapping quality (MQ) and mapping quality rank sum tests (MQRankSum). Variant calling was performed using the Genome Analysis Toolkit (GATK) HaplotypeCaller (v3.2.2) in discovery mode. Parameters included a minimum base quality score of 10 and a minimum mapping quality score of 20, with coverage downsampled to a maximum of 250×. Variants were emitted at a minimum confidence threshold of 5.0 and called at 10.0. Indel modeling was set to conservative mode, with a maximum of six alternate alleles considered per site. Variants were annotated and filtered using the Golden Helix VarSeq software package (Golden Helix, Inc., Bozeman, MT, https://www.goldenhelix.com/products/VarSeq/). Annotation included evaluation against the ClinVar database to identify previously reported pathogenic variants. In total, approximately 30,000 exome variants were annotated, and pathogenic calls were prioritized for downstream interpretation. Variants not meeting minimum quality thresholds were filtered out.

For P2, DNA extracted from whole blood in a clinical lab. Sequencing data were processed and analyzed following standard next-generation sequencing (NGS) practices. Paired-end reads were aligned to the human reference genome (hg19) using BWA-MEM v0.7.9a-r786 with default parameters and read group assignment. Post-alignment processing included duplicate marking with Picard MarkDuplicates v1.111 and local realignment around indels with GATK IndelRealigner v3.1.7, using the Mills and 1000G gold standard indel set as a reference. Reads were subsequently processed with GATK PrintReads before conversion to CRAM format with samtools v1.22.1. The reference genome sequence dictionary contained canonical chromosomes, alternate haplotypes, unplaced contigs, and the mitochondrial genome. Variant calling was performed using samtools v0.1.18, producing VCF files (v4.1) annotated with depth, mapping quality, allele frequency estimates, genotype likelihoods, Hardy–Weinberg equilibrium statistics, and bias metrics (e.g., strand, base quality, mapping quality, and tail distance bias). Genotype-level fields included genotype calls, genotype quality, depth, likelihoods, and strand bias metrics. This workflow provided high-confidence single-nucleotide variant (SNV) and indel calls for downstream interpretation.

References

[1] P.D. Stenson, M. Mort, E.V. Ball, K. Shaw, A. Phillips, and D.N. Cooper, The Human Gene Mutation Database: building a comprehensive mutation repository for clinical and molecular genetics, diagnostic testing and personalized genomic medicine. Hum Genet 133 (2014) 1-9.

[2] J.T. Robinson, H. Thorvaldsdottir, W. Winckler, M. Guttman, E.S. Lander, G. Getz, and J.P. Mesirov, Integrative genomics viewer. Nat Biotechnol 29 (2011) 24-6.
